# Low Intra-Individual Variation in Mean Platelet Volume Over Time in Systemic Lupus Erythematosus

**DOI:** 10.3389/fmed.2021.638750

**Published:** 2021-04-20

**Authors:** Lina Wirestam, Birgitta Gullstrand, Andreas Jern, Andreas Jönsen, Petrus Linge, Helena Tydén, Robin Kahn, Anders A. Bengtsson

**Affiliations:** ^1^Section of Rheumatology, Department of Clinical Sciences Lund, Lund University, Lund, Sweden; ^2^Section of Pediatrics, Department of Clinical Sciences Lund, Lund University, Lund, Sweden; ^3^Wallenberg Centre of Molecular Medicine, Lund University, Lund, Sweden

**Keywords:** mean platelet volume, systemic lupus erythematosus, autoimmunity, platelets, biomarkers

## Abstract

Platelets have recently emerged as important immune modulators in systemic lupus erythematosus (SLE), in addition to their role in thrombosis and cardiovascular disease. However, studies investigating mean platelet volume (MPV) in SLE are often scarce, conflicting and cross-sectional. In this study, MPV was measured in clinical routine throughout a defined time-period to quantify both individual MPV fluctuations and investigate if such variations are associated with disease activity and clinical phenotypes of SLE. Of our 212 patients, 34 patients had only one MPV value reported with the remaining 178 patients having between 2 and 19 visits with recorded MPV values. The intra-individual MPV variation was low, with a median variation of 0.7 fL. This was further supported by the finding that 84% of patients stayed within their reference interval category (i.e., small, normal or large) over time. In our cohort, no correlation between disease activity and MPV neither cross-sectionally nor longitudinally was found. Mean platelet volume values were significantly smaller in SLE patients (mean 10.5 fL) compared to controls (mean 10.8 fL), *p* < 0.0001. Based on the reference interval, 2.4% (*n* = 5) of patients had large-sized platelets, 84.4% (*n* = 179) had normal-sized and 13.2% (*n* = 28) had small-sized. A larger proportion (85.7%) of patients with small-sized platelets met the anti-dsDNA criterion (ACR10b; *p* = 0.003) compared to patients with normal and large (57.6%) sized platelets. In conclusion, the intra-individual MPV variation was of low magnitude and fluctuations in disease activity did not have any significant impact on MPV longitudinally. This lack of variability in MPV over time indicates that measuring MPV at any time-point is sufficient. Further studies are warranted to evaluate MPV as a possible biomarker in SLE, as well as to determine the underlying mechanisms influencing platelet size in SLE.

## Introduction

Accumulation of autoantibodies and immune complexes leading to activation of type I interferon (IFN) signaling are central in the pathogenesis of systemic lupus erythematosus (SLE) (1). SLE is characterized as a chronic heterogenous autoimmune disease whereby variable clinical and serological presentation can be seen between patients and across disease stages. Secondary antiphospholipid syndrome (APS) occurs in ~20–30% of SLE patients, typically manifesting in individuals through thrombosis and pregnancy complications, with the clinical spectrum also including thrombocytopenia (2, 3). The role of platelets in hemostasis and thrombosis is well-established. However, in recent decades, platelets have been recognized as a part of the innate immune system, with the complexity of platelets becoming more evident (4, 5). Platelets inherit various components from megakaryocytes such as mRNA, proteins and cytoplasmic organelles, including mitochondria and granules. Platelets may also synthesize new proteins from their preformed mRNA and are attributed to having functional apoptosis and even cell division (6). Finally, expression of several adhesion molecules, e.g., integrins, which mediate direct cell-cell interactions, can also be performed by platelets. Hence, such cells are able to bind both pathogens and immune cells and are being increasingly recognized as important mediators in SLE because of their immune modulating capacity (5).

Platelet indices can easily be measured by automated hematological analyzers. The mean platelet volume (MPV), defined as the average size of circulating platelets, is often calculated through impedance technology, whereby every pulse is counted and the pulse size is accumulated. The total pulse volume is then divided by the pulse count to yield the MPV. During physiological conditions, there is an inverse non-linear relationship between the MPV and platelet count (7). However, in disorders with increased platelet destruction, such as immune thrombocytopenic purpura (ITP), the mean platelet volume is increased (8).

Research investigating MPV in SLE are often scarce and conflicting. Despite studies observing a lower platelet size in SLE patients compared to healthy individuals (9, 10), increased MPV may also act as an early indicator of reactivation within juvenile SLE (11). Furthermore, there is inconsistent data regarding the association between MPV and disease activity (11–15). For example, a meta-analysis by Zhao et al. from 2018 could not find any difference in MPV between active and inactive SLE patients (16). Additionally, increased MPV has been associated with augmented platelet reactivity and is suggested to be a predictor of thrombosis and cardiovascular risk in non-SLE (17). This hypothesis aligns with data highlighting an increased MPV found in patients with both primary and secondary APS, especially for such individuals who were triple positive for anti-cardiolipin, anti-b2GP1 and lupus anti-coagulant (18, 19). However, this contrasts to previous findings by our group, where patients with secondary APS displayed decreased MPV (10).

Little is known about the underlying mechanisms that may influence platelet size in SLE. Platelets may undergo apoptosis and thereby decrease in size (5, 20). A general increase in apoptosis rate is typically observed in SLE (21), with platelets from SLE patients displaying ultrastructural changes such as blebbing (22). However, the role of platelet apoptosis has not yet been explored in SLE. Other possible explanations for a decreased platelet size could either be an increased consumption of large platelets at inflammation sites (23) and/or an overproduction of proinflammatory cytokines and acute phase proteins which may interfere with megakaryopoiesis (24, 25). It is currently unknown if MPV remains constant or fluctuates in SLE patients, or if having small platelets could represent a clinical phenotype.

Earlier studies by us and others examining MPV in SLE have cross-sectional study designs. Longitudinal studies are warranted where it can be determined if MPV fluctuate or remain rather constant. This may aid in evaluating MPV as a possible biomarker for disease activity and/or specific clinical phenotypes within SLE. It may also give better insight into the role of platelets in SLE immunopathogenesis. This is, to our knowledge, the first study aiming to analyze longitudinal MPV values and characterize MPV fluctuations while investigating potential associations with disease activity and clinical phenotypes to better define the role of MPV in SLE.

## Materials and Methods

### Study Population

Clinical patient characteristics (*n* = 212) are outlined in Table 1. All patients included met at least 4 of 11 American College of Rheumatology (ACR) classification criteria (26). Data was collected from SLE patients taking part in our prospective follow-up program at the department of Rheumatology in Lund. Clinical routine analyses [C-reactive protein (CRP), erythrocyte sedimentation rate (ESR), leukocyte variables, anti-dsDNA, complement components] and recording of disease activity, organ damage and medications are registered at each visit. Disease activity is assessed using the Systemic Lupus Erythematosus Disease Activity Index 2000 (SLEDAI-2K) (27) and organ damage is evaluated by the SLICC/ACR damage index (SDI) (28). Distribution of disease activity and medications used are outlined in Table 2. Mean platelet volume (in EDTA) measurements were performed through impedance methodology using a Sysmex XN-10 analyzer (Sysmex, Kobe, Japan) between October 2013 to January 2020, and included all routine follow-up data since 2016 at the Department of Clinical Chemistry at Lund University Hospital. Normal reference (*n* = 2,345, 1,916 men, and 429 women) values of MPV were received from the Department of Clinical Chemistry at Lund University Hospital. The study was approved by The Central Ethical Review Board of Lund University (Dnr 2010/668) and informed consent was obtained from all participants according to the Declaration of Helsinki.

**Table 1:** Clinical characteristics of the SLE patients (*n* = 212).

Age, median (range), years	56 (24–91)
Females, percent, (*n*)	88 (186)
ACR criteria, median (range)	6 (4–10)
Malar rash, percent, (*n*)	53 (112)
Discoid rash	29 (61)
Photosensitivity	63 (133)
Oral ulcers	32 (67)
Arthritis	81 (171)
Serositis	49 (103)
Renal disease	35 (74)
Neurological disorder	8 (17)
Hematological manifestations	65 (137)
Leukopenia	40 (85)
Lymphopenia	37 (79)
Thrombocytopenia	23 (48)
Immunology	67 (141)
Anti-dsDNA antibodies	61 (130)
ANA	99 (210)
SLICC/ACR-DI score, median (range)	1 (0–8)
Ocular, percent (*n*)	15 (32)
Neuropsychiatric	19 (41)
Renal	6 (12)
Pulmonary	7 (15)
Cardiovascular	11 (24)
Peripheral vascular	12 (26)
Gastrointestinal	6 (12)
Musculoskeletal	16 (34)
Skin	10 (22)
Pre-mature gonadal failure	0.5 (1)
Diabetes	2 (4)
Malignancy	7 (15)

**Table 2:** Distribution of disease activity and medications.

**SLEDAI distribution of all visits in all patients, percent (*n*)**	
0–3	70 (665)
4–9	23 (215)
≥10	3 (28)
Missing	4 (36)
**SLEDAI distribution within individual patients, percent (*n*)**	
0–3	61.8 (131)
4–9	34 (72)
≥10	3.8 (8)
Missing	0.4 (1)
Patients with flares^*^, percent (*n*)	13.7 (29)
**SLEDAI descriptors ever during study period, percent (*n*)**	
Seizure	0 (0)
Psychosis	0 (0)
Organic brain syndrome	0 (0)
Visual disturbance	0 (0)
Cranial nerve disorder	0 (0)
Lupus headache	0.9 (2)
Cerebrovascular accident	0 (0)
Vasculitis	3 (6)
Arthritis	11 (23)
Myositis	0 (0)
Urinary casts	0 (0)
Hematuria	2 (4)
Proteinuria	4 (8)
Pyuria	1.4 (3)
Rash	18 (39)
Alopecia	7.5 (16)
Mucosal ulcers	4 (9)
Pleurisy	1.4 (3)
Pericarditis	0 (0)
Low complement	53 (113)
Increased DNA binding	31 (65)
Fever	2.4 (5)
Thrombocytopenia	1.4 (3)
Leukopenia	6 (12)
**Medication ever during study period, percent (*n*)**	
Corticosteroids	61 (130)
Hydroxychloroquine	71 (150)
Methotrexate	10 (21)
Azathioprine	22 (47)
Ciclosporin	2 (5)
Mycophenolate mofetil	15 (31)
Belimumab	7 (15)
Rituximab	4 (9)
Intravenous immune globulin therapy	1 (2)

* *Flares are defined as a SLEDAI-increase of >4*.

### Statistical Analyses

Spearman's rank correlation was used to determine possible associations between MPV and disease activity markers and between MPV and age. Associations between MPV and different disease phenotypes and additionally with damage was evaluated by Chi-Square-testing. To compare SLE and control MPV values, a *t*-test was used where mean values, standard deviation and the number of observations were reported. Mann-Whitney U-testing was used to compare MPV values between men and women. In order to examine the possible influence of disease activity, different clinical phenotypes and medications on MPV longitudinally, we used generalized estimating equations (GEE) with MPV as the dependent variable. Statistical significance was set at *p* < 0.05, along with 95% CI. Statistical analyses were done in SPSS Statistics v.25 (IBM, Amonk, NY, USA) or GraphPad Prism, version 7.0 (GraphPad Software, San Diego, CA, USA).

## Results

### Distribution of Mean Platelet Volume in SLE

**Figure 1:**
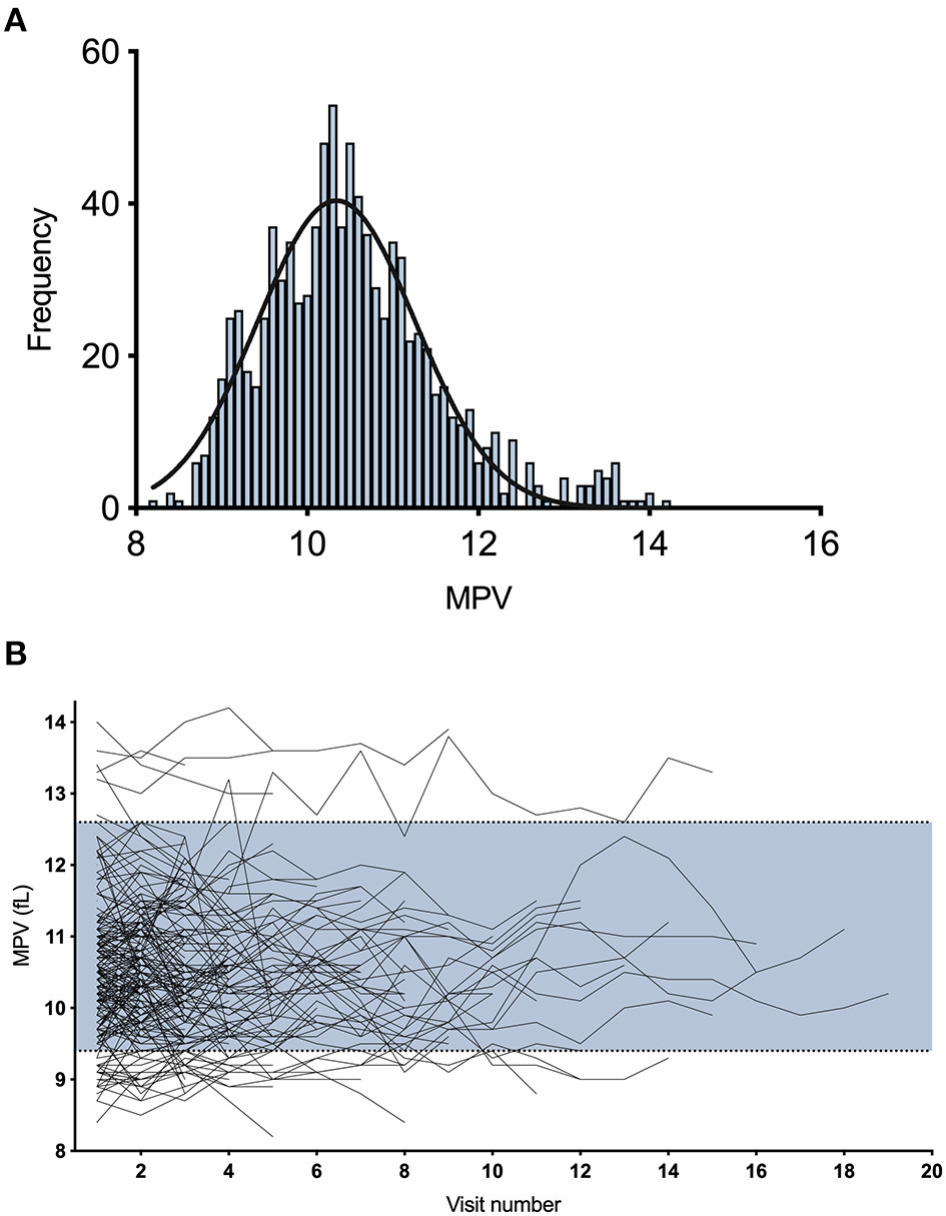
Distribution of mean platelet volume in SLE measured longitudinally. **(A)** Frequency distribution of all 212 study patients collected during 944 visits with a fitted normal curve. **(B)** Individual variations in MPV over time. The blue area represents the normal range (9.4–12.6 fL).

During the study period, there were 212 SLE-patients with MPV values. Of the 212 patients examined, 34 had only one MPV value reported. The remaining 178 patients had between 2 and 19 visits (mean 5 visits, median 4 visits) where MPV values were analyzed, resulting in a total of 944 visits. The distribution of all MPV values is illustrated in Figure 1A. Mean platelet volume values ranged from 8.2 to 14.2 fL with a median of 10.4 fL (95% CI 10.25–10.5). Individual variations are illustrated in Figure 1B.

### Low Intra-Individual Variation in MPV Over Time

**Figure 2:**
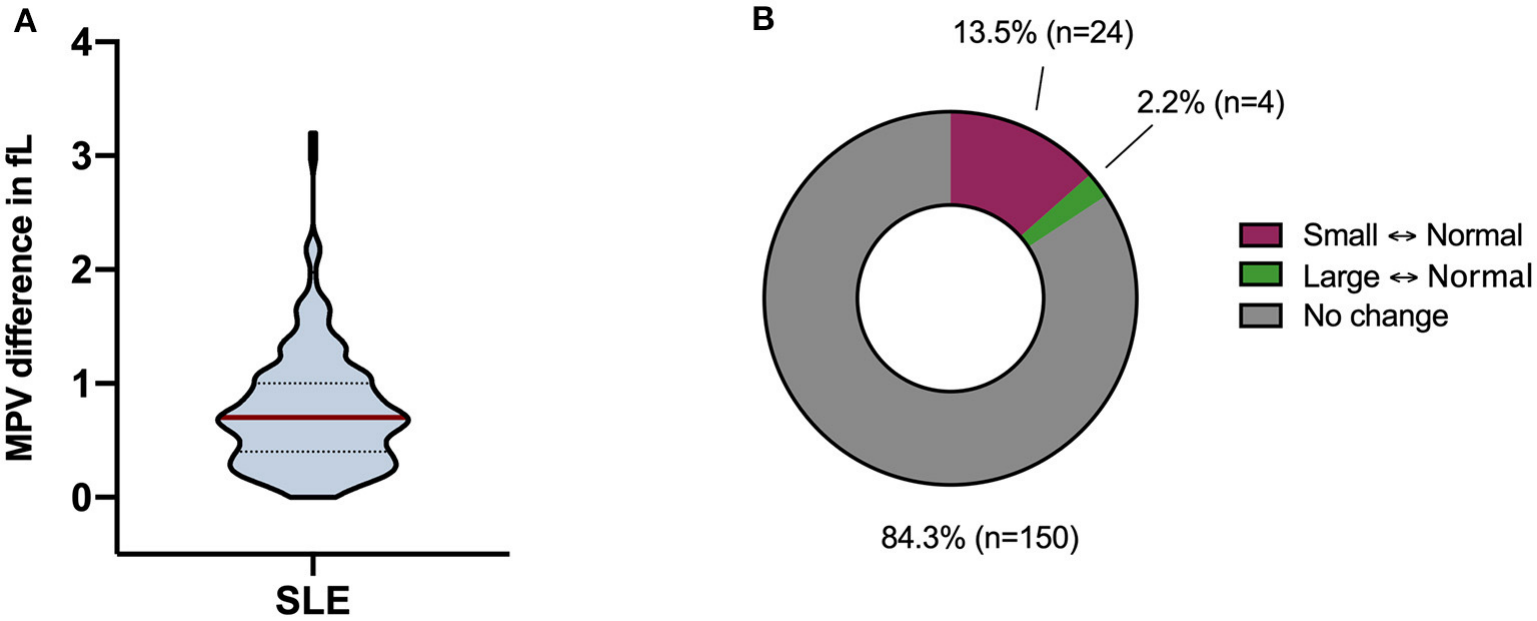
Mean platelet volume (MPV) fluctuations. **(A)** Differences in MPV over time were calculated for each patient and illustrated through a violin plot. The MPV varied with a median of 0.7 fL (range 0–3.2 fL). The red line represents the median and the dotted lines represent the quartiles. **(B)** Transfer between the reference interval groups (i.e., small, normal, and large) longitudinally. Twenty-four (13.5%) patients changed between normal and small, and 4 (2.2%) patients shifted between normal and large. The rest of the patients (84.3%, *n* = 150) stayed within their reference interval group over time.

In order to characterize the fluctuation in MPV values, we calculated the femtolitre (fL) difference for each patient (Figure 2A). The MPV varied with a median of 0.7 fL (95% CI 0.6–0.7) (range 0–3.2 fL). We also calculated the standard deviation for each patient with more than three visits. The standard deviation of MPV values ranged from 0.08 to 1.2 with a median of 0.32 fL (95% CI 0.29–0.36). The intra-individual biological variation for MPV had a median of 2.9% (95% CI 2.6–3.1). To further segregate MPV variations, we categorized patients into “small,” “normal,” or “large” size MPV categories based on the reference interval (9.4–12.6 fL) from the Department of Clinical Chemistry at Lund University Hospital and investigated how patients shift between the three groups (Figure 2B). Of all investigated patients, 28 (15.7%) shifted between the three different reference interval groups. Among those who shifted, 24 patients changed between normal and small, with 4 patients shifting between normal and large. Most of the patients (84.3%, *n* = 150) stayed within their reference interval group during the study period. Thus, we were able to quantify the change in MPV over time and conclude that this change was of low magnitude.

### Analyses of MPV Over Time and Clinical Variables

**Figure 3:**
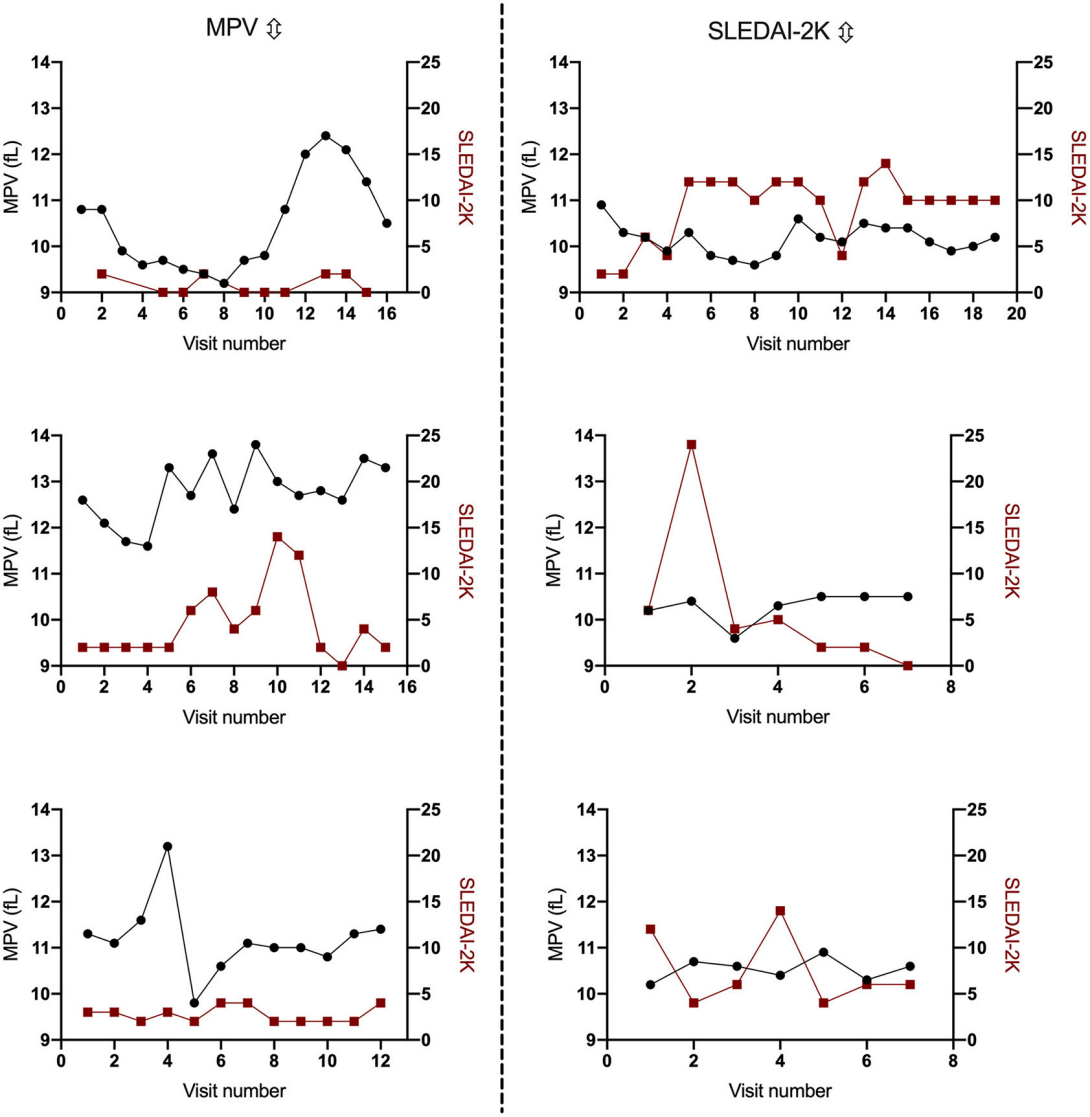
Examples of patients with larger fluctuations in mean platelet volume (MPV) or disease activity (SLEDAI-2K). The left three graphs illustrate patients with larger MPV fluctuations and the right three graphs illustrate patients with larger fluctuations in disease activity.

We found no correlation between SLEDAI-2K and mean MPV (cross-sectional; *r* = 0.056, *p* = 0.438). To further examine the possible influence of SLEDAI-2K longitudinally on MPV we used GEE. SLEDAI-2K was not associated with MPV in GEE, either when analyzed as a continuous variable (*B* = 0.001, 95% Wald CI −0.042–0.044, *p* = 0.968), nor as a bivariate variable when a cut-off of >4 was set for active disease (*B* = 0.152, 95% Wald CI −0.138–0.442, *p* = 0.303). The SLEDAI-2K descriptors were also analyzed individually and were found to have no influence on MPV. Sub-analyses were also conducted between MPV and SLEDAI-2K in thrombocytopenic patients (patients fulfilling ACR 9c; *B* = 0.042, 95% Wald CI −0.033–0.117, *p* = 0.276) and in patients with secondary APS (*B* = 0.022, 95% Wald CI −0.078–0.121, *p* = 0.665) but SLEDAI-2K did not influence MPV in any of these subgroups. Regarding anti-dsDNA, 8% of the patients (*n* = 17) were positive at every follow-up, 69% (*n* = 147) were negative and 23% (*n* = 48) alternated. As described above, changes of MPV over time were very low and not related to SLEDAI on a group-level. However, in a few individuals, we observed MPV variations over time, with Figure 3 highlighting three patients with the largest variations over time using MPV-values and SLEDAI. Herein, three additional patients were also selected through visitation number and large variation size in SLEDAI. As seen in Figure 3, individual MPV values may vary over time but there were no covariations with SLEDAI-2K. Additionally, no associations were found between MPV and other inflammatory markers, i.e., CRP (*B* = −0.009, 95% Wald CI −0.027–0.009, *p* = 0.306) and ESR (*B* = −0.001, 95% Wald CI −0.009–0.008, *p* = 868). However, an increased number of fulfilled ACR criteria had a modest impact on MPV-variations (*B* = −0.116, 95% Wald CI −0.228 to −0.004, *p* = 0.042). This suggests that for every added number of fulfilled ACR criteria, the MPV decreases by 0.116 fL. We also investigated if there were any associations between organ damage (SDI) as a continuous variable and MPV-variations (*B* = −0.058, 95% Wald CI −0.134–0.018, *p* = 0.133), but found no associations.

Any documented positivity for different autoantibodies since diagnosis (i.e., anti-cardiolipin, anti-b2GPI, anti-C1q, anti-ribosomal protein P) did not influence MPV over time, nor did levels of complement C3, C4, or C1q (Table 3). Notably, being diagnosed with secondary APS, did not have any impact on MPV longitudinally (*B* = −0.109, 95% Wald CI −0.481–0.262, *p* = 0.564).

**Table 3:** Impact of disease activity, autoantibodies, complement levels, and medications on mean platelet volume (MPV) variations, analyzed by generalized estimating equations (GEE).

	***B***	**95% Wald CI**	***p-*value**
**SLEDAI descriptors**			
Seizure	–		
Psychosis	–		
Organic brain syndrome	–		
Visual disturbance	–		
Cranial nerve disorder	–		
Lupus headache	0.172	−0.196−0.540	0.359
Cerebrovascular accident	–		
Vasculitis	−0.784	−1.242−0.327	**0.001**
Arthritis	−0.297	−0.851−0.256	0.292
Myositis	–		
Urinary casts	–		
Hematuria	1.016	0.861−1.172	** <0.001**
Proteinuria	−0.386	−0.889−0.117	0.132
Pyuria	−0.254	−0.712−0.204	0.278
Rash	−0.172	−0.445−1.000	0.215
Alopecia	0.347	−0.330−1.024	0.315
Mucosal ulcers	0.042	−0.492−0.576	0.878
Pleurisy	0.022	−0.289−0.333	0.891
Pericarditis	–		
Low complement	0.158	−0.140−0.456	0.300
Increased DNA binding	0.150	−0.183−0.484	0.377
Fever	−0.448	−1.130−0.235	0.199
Thrombocytopenia	1.216	0.163−2.269	0.024
Leukopenia	0.387	−0.053−0.827	0.085
**Autoantibodies**			
Anti-cardiolipin	−0.158	−0.596−0.279	0.479
Anti-b2GPI	−0.233	−0.753−0.288	0.381
Anti-C1q	−0.311	−0.892−0.269	0.293
Anti-ribosomal protein P	0.299	−0.479−1.078	0.451
**Complement levels**			
C3	−0.108	−0.295−0.079	0.256
C4	−0.209	−2.940−2.522	0.881
C1q	−0.003	−0.013−0.008	0.628
**Medications**			
Corticosteroids	−0.041	−0.355−0.273	0.800
Hydroxychloroquine	0.094	−0.233−0.421	0.572
Methotrexate	0.031	−0.374−0.437	0.879
Azathioprine	−0.361	−0.708−0.013	**0.042**
Ciclosporin	0.279	−0.788−1.346	0.609
Mycophenolate mofetil	0.113	−0.399−0.625	0.665
Belimumab	0.074	−0.534−0.681	0.812
Rituximab	−0.083	−0.773−0.607	0.814
Intravenous immune globulin therapy	0.615	−0.382−1.612	0.226

*Bold values indicates that they are statistically significant*.

We also analyzed the impact of different ongoing medications (corticosteroids, hydroxychloroquine, methotrexate, azathioprine, ciclosporin, mycophenolate mofetil, belimumab, rituximab, and intravenous immune globulin therapy) on MPV values (Table 3). Notably, azathioprine was found to be associated with a lower MPV, (*B* = −0.361, 95% Wald CI 0.013–0.708, *p* = 0.042). However, we were unfortunately unable to analyze the impact of NSAIDs and antiplatelet drugs due to missing data.

### Cross-Sectional MPV Analyses

**Figure 4:**
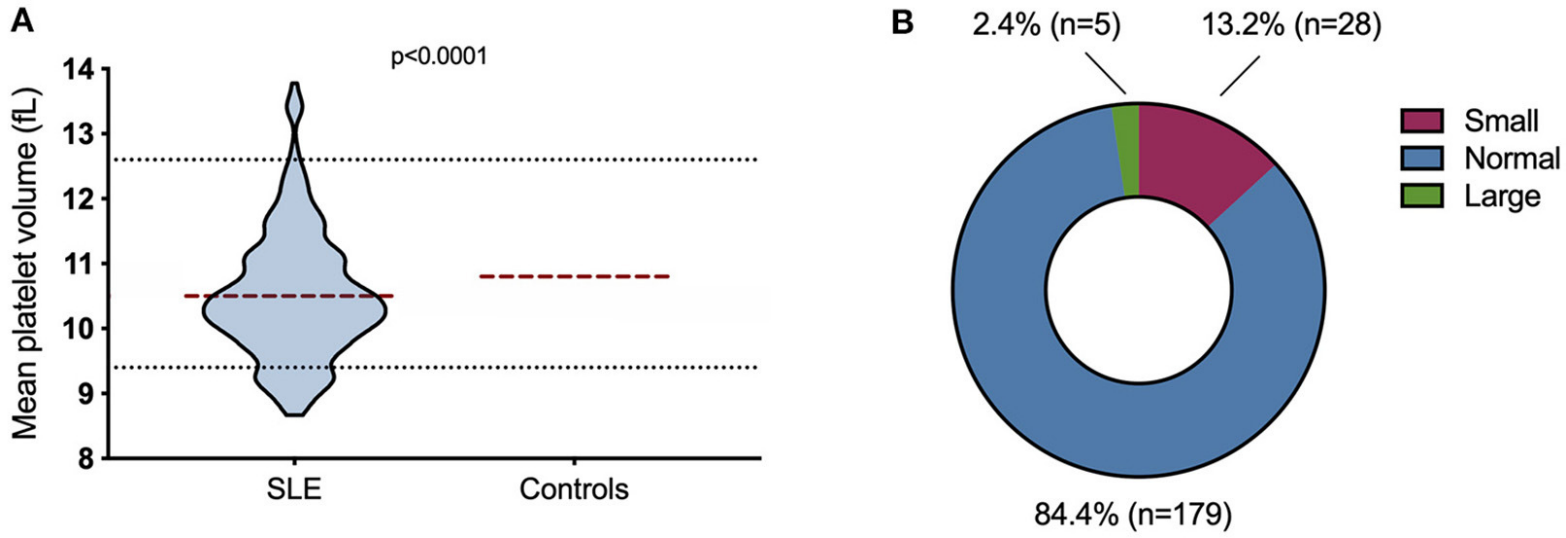
Mean platelet volume (MPV) in SLE patients. **(A)** The MPV ranged from 8.7 to 13.8 fL (*n* = 212). The dotted lines represent the normal range (9.4–12.6 fL). The red line represents the mean; 10.5 fL for SLE and 10.8 fL for controls. **(B)** MPV-values were grouped into “small,” “normal,” or “large” size MPV based on the reference interval. 2.5% (*n* = 5) of the patients were large, 84.4% (*n* = 179) were normal and 13.2% (*n* = 28) were small.

As it was concluded that variations of MPV over time were very low, cross-sectional analyses were subsequently performed. We calculated the mean MPV value for all SLE patients (Figure 4A), which ranged from 8.7 to 13.8 femtolitres (fL). The reference range calculated by Sysmex ranges from 9.4 to 12.6 fL, with a mean value of 10.8 fL and a standard deviation of 0.8 fL. Mean platelet volume was significantly smaller in SLE patients (mean value: 10.5 fL; 95% CI: 10.25–10.5 fL) compared to controls (mean value: 10.8 fL, 95% CI: 9.4–12.6 fL, *p* < 0.0001). To further examine MPV within SLE, we categorized patients into “small,” “normal,” or “large” size MPV categories, based on the reference interval (9.4–12.6 fL). Patients with MPV values of <9.4 fL were considered as small, between 9.4 and 12.6 fL normal and >12.6 fL large. 2.4% (*n* = 5) of patients had large platelet size, 84.4% (*n* = 179) had normal and 13.2% (*n* = 28) had small (Figure 4B). We found no correlation between MPV and age (*r* = 0.058, *p* = 0.403), nor any differences between males and females [median: 10.49 (males), 10.40 (women), *p* = 0.75]. However, an inverse correlation between MPV and platelet count (*r* = −0.365, *p* < 0.0001) was detected.

We further evaluated associations between MPV and different disease phenotypes defined as fulfilled ACR classification criteria (Table 4). A larger proportion of patients with a small mean MPV met the anti-dsDNA criterion (ACR10b) compared to patients with normal and large mean MPVs, [85.7% (*n* = 24) compared to 57.6% (*n* = 106), *p* = 0.003]. Patients who developed damage did not show any differences in MPV, including when analyzing specific organ domains of the SDI (Table 4).

**Table 4:** Analyses of categorized MPV-values with disease phenotypes defined as fulfilled ACR classification criteria and organ damage.

**Item, % (*n*)**	**Small (*n* = 28)**	**Normal and large (*n* = 184)**	***p-*value**
**ACR criteria**			
1. Malar rash	57.1 (16)	52.2 (96)	0.388
2. Discoid rash	32.1 (9)	28.3 (52)	0.413
3. Photosensitivity	64.3 (18)	62.5 (115)	0.516
4. Oral ulcers	32.1 (9)	31.5 (58)	0.553
5. Arthritis	82.1 (23)	80.4 (148)	0.533
6. Serositis	50 (14)	48.4 (89)	0.516
6a. Pleuritis	46.4 (13)	40.2 (74)	0.336
6b. Pericarditis	10.7 (3)	19 (35)	0.216
7. Renal disorder	32.1 (9)	35.3 (65)	0.460
8. Neurologic disorder	10.7 (3)	7.6 (14)	0.395
8a. Seizures	10.7 (3)	4.3 (8)	0.164
8b. Psychosis	0 (0)	3.3 (6)	0.423
9. Hematologic disorder	60.7 (17)	65.2 (120)	0.395
9a. Hemolytic anemia	3.6 (1)	7.1 (13)	0.423
9b. Leukopenia	42.9 (12)	39.7 (73)	0.451
9c. Lymphopenia	32.1 (9)	38 (70)	0.352
9d. Thrombocytopenia	25 (7)	22.3 (41)	0.456
10. Immunologic disorder	85.7 (24)	63.6 (117)	**0.014**
10a. positive LE cell	3.6 (1 st)	1.6 (3)	0.435
10b. Anti-DNA	85.7 (24 st)	57.6 (106)	**0.003**
10c. Anti-Sm	14.3 (4 st)	11.4 (21)	0.427
10d. False positive syphilis test	0 (0 st)	6.5 (12)	0.174
11. ANA	100 (28 st)	98.9 (182)	0.753
**SLICC/ACR-DI**			
Damage ever	67.9 (19)	58.4 (101)	0.409
Ocular	25 (7)	14.4 (25)	0.166
Neuropsychiatric	25 (7)	19.5 (34)	0.612
Renal	7.1 (2)	5.7 (10)	0.674
Pulmonary	3.6 (1)	8 (14)	0.699
Cardiovascular	17.9 (5)	10.9 (19)	0.341
Peripheral vascular	14.3 (4)	12.6 (22)	0.765
Gastrointestinal	7.1 (2)	5.7 (10)	0.674
Musculoskeletal	28.6 (8)	14.9 (26)	0.099
Skin	17.9 (5)	9.8 (17)	0.200
Pre-mature gonadal failure	0 (0)	0.6 (1)	1.000
Diabetes	0 (0)	2.3 (4)	1.000
Malignancy	10.7 (3)	6.9 (12)	0.445

*Bold values indicates that they are statistically significant*.

## Discussion

Despite a knowledge that platelets may alter their phenotype, function and size within different disease states, studies investigating MPV in SLE are scarce, conflicting and cross-sectional. However, results from longitudinal studies may shed light on potential MPV fluctuations and facilitate the interpretation of cross-sectional studies. This is, to our knowledge, the first study investigating MPV longitudinally in relation to SLE disease manifestations, activity, medication and organ damage.

A major finding noted that intra-individual variation was stable over time, with a median standard deviation for all patients being 0.32 fL. This is further supported by a large majority of the patients, (84%) were found to stay within their reference interval group (i.e., small, normal, or large) over time. Other studies examining MPV longitudinally though rare, also point in the same direction. The prognostic role of MPV within cirrhosis patients has additionally been studied. Mean platelet volume was measured five times during a period of 12 months and MPV turned out as quite stable (29). In healthy individuals, the intra-individual biological variation for MPV has been reported as 2.6% (30), compared to 2.9% in our study. Moreover, a cross-sectional population-based study shows that MPV remains stable over a lifetime, and does not correlate with age (31).

In SLE, a decreased MPV (12–14), as well as an increased MPV (11, 15) have been both reported in patients with active disease. However, a meta-analysis by Zhao et al. (16) could not find any differences in MPV between active and inactive SLE patients. We additionally could not find any correlation between disease activity and MPV when investigating our patient cohort cross-sectionally, nor did the disease activity have any significant impact on MPV longitudinally. Notably, many of our patients included have low disease activity.

Platelet size may be affected by epigenetic influences and environmental factors such as air pollutions (32). Heritability studies have shown that MPV is influenced by genetic factors. There are both genome wide association and whole exome sequencing studies that have identified genes influencing the cytoskeleton, signaling proteins, membrane proteins, megakaryocyte development and platelet production (33–35). As no MPV variations over time despite a general decrease in MPV were observed, influence due to genetic factors may be speculated. It has been hypothesized that MPV may be influenced by sex and age, but no conclusive data has been presented to date (31, 36–38). We found no correlations between MPV and sex, or with age. Regardless of sex and age, there is a wide variability of MPV in healthy subjects, resulting in a large normal range. Previous observations by us have showed lower MPV values in SLE patients (10), and herein we report that the MPV is significantly smaller within SLE patients compared to controls. These two studies also contain overlapping patient cohorts. Furthermore, 13.2% of our patients are below the normal range and thus grouped as small. It should be noted that large and small platelets within the same individual can differ from both a phenotype and functional perspective (39). Small platelet mRNA is linked to apoptosis and cell death regulation, with smaller platelets showing a higher abundance of immunoglobulins, inflammatory proteins and apolipoproteins (39, 40). Lack of association between MPV-variations and disease activity could possibly be due to the ongoing chronic long-standing inflammation, which is known to be of major importance in SLE.

Since MPV appeared stable over time, we continued with cross-sectional analyses. We evaluated possible associations of MPV with different disease phenotypes (i.e., fulfilled ACR criteria) and found that a larger proportion of patients with small platelets met the anti-dsDNA criterion. This observation may be related to findings suggested that platelets could be activated by DNA-containing immune complexes *via* FcγRIIA or by free anti-DNA antibodies resulting in morphological changes and release of microparticles (41, 42). Furthermore, anti-dsDNA and/or anti-dsDNA-containing immune complexes may induce degranulation of secretory α-granules and dense granule with serotonin (43), and we have previously reported decreased serotonin levels in SLE platelets (44). Platelet activation, degranulation and microparticle formation induced by anti-dsDNA are thus examples of possible mechanistic explanations that may contribute to a decreased platelet size (10). Such structural and functional changes by anti-dsDNA antibodies may promote a pro-thrombotic state in SLE, but also contribute to dysregulated immune reactions.

Large MPV has been associated with platelet reactivity and proposed as a predictor of thrombosis and cardiovascular risk (17). Patients with primary and secondary APS have been previously reported to have increased MPV (18, 19). We have also previously described an association between low platelet size, determined by the median forward scatter of isolated platelets measured by flow cytometry, and secondary APS in SLE patients (10). In the present investigation, being diagnosed with secondary APS had no impact on MPV longitudinally, measured by impedance methodology, in our GEE model. Increased MPV has been observed in different disease states with organ damage e.g., cardiovascular diseases, cerebral stroke, respiratory diseases and chronic renal failure [reviewed in (45)]. We found no association between organ damage (SDI) and MPV in our SLE patients. The relatively small number of damage events most likely reflects well-controlled patients but likewise generates uncertainties. A longer follow-up with a larger population size may clarify whether MPV is related to organ damage.

There are many pre-analytical and intra-analytical parameters that can affect platelet size. Hematology analyzers used in routine diagnostics use optical light scatter or impedance counting to measure MPV. Thus, it is important to remember that the measurement principles used can influence results and aggravate comparisons between different analyzers (46, 47). Moreover, anticoagulants used and the time frame from blood sampling to analysis additionally affect MPV. For example, EDTA, used in our study, is a better suited anticoagulant for MPV measurement compared to sodium citrate and heparin, which have both proved to be unreliable in the measurement of platelet volume (48). To fully compare clinical studies, the type of anticoagulant and time frame from blood sampling to analysis must be standardized.

Limitations of this study include missing data regarding NSAIDs, antiplatelet drugs and anti-depressants belonging to the group of selective serotonin reuptake inhibitors (SSRI), which are drugs with known effects on platelet function and could theoretically affect MPV. Moreover, the relatively small number of patients with high-disease activity and flares most likely reflect well-controlled SLE patients representing the situation of today in developed countries. However, we cannot exclude that different patient selections may have generated different results.

In conclusion, the intra-individual MPV variation over time was of low magnitude and fluctuations in disease activity had no significant impact on MPV measured longitudinally. This lack of MPV variability over time indicates that measuring MPV at any timepoint is sufficient. Furthermore, studies of MPV with cross-sectional design could lead us further in determining the role of MPV as a possible biomarker in SLE. We observed a decreased MPV in SLE, with it being especially pronounced in patients with anti-dsDNA antibodies. Further studies are warranted to evaluate MPV as a possible biomarker in SLE, and our observations need to be confirmed in independent cohorts, as well-determining the underlying mechanisms influencing platelet size in SLE.

## Data Availability Statement

The original contributions presented in the study are included in the article/supplementary material, further inquiries can be directed to the corresponding author.

## Ethics Statement

The studies involving human participants were reviewed and approved by The Central Ethical Review Board of Lund University (Dnr 2010/668). The patients/participants provided their written informed consent to participate in this study.

## Author Contributions

LW acquisition and analyses of patient data, interpretation of results, and writing of the manuscript. BG, AJe, AJö, PL, HT, and RK interpretation of results and writing of the manuscript. AB designing of the project, interpretation of results, and writing of the manuscript. All authors contributed to the article and approved the submitted version.

## Conflict of Interest

The authors declare that the research was conducted in the absence of any commercial or financial relationships that could be construed as a potential conflict of interest.
